# COP9 signalosome complex is a prognostic biomarker and corresponds with immune infiltration in hepatocellular carcinoma

**DOI:** 10.18632/aging.205646

**Published:** 2024-03-11

**Authors:** Jiahui Liu, Dexing Han, Junfeng Xuan, Jinye Xie, Weijia Wang, Quan Zhou, Kang Chen

**Affiliations:** 1 Department of Clinical Laboratory, Zhongshan City People’s Hospital, The Affiliated Zhongshan Hospital of Sun Yat-Sen University, Zhongshan 528400, Guangdong, China; 2 Laboratory of Basic Medical Science, General Hospital of Southern Theater Command of PLA, Guangzhou 510000, Guangdong, China; 3 The Second Clinical Medical College, Guangzhou University of Chinese Medicine, Guangzhou 510000, Guangdong, China; 4 Research Centre of Basic Integrative Medicine, School of Basic Medical Sciences, Guangzhou University of Chinese Medicine, Guangzhou 510000, Guangdong, China

**Keywords:** COP9 signalosome, hepatocellular carcinoma, prognosis, immune infiltration, therapeutic target

## Abstract

Hepatocellular carcinoma (HCC) is among the most common deadly tumors but still lacks specific biomarkers for diagnosis, prognosis, and treatment guidance. The COP9 signalosome (COPS) is an essential regulator of the ubiquitin conjugation pathway upregulated in various cancers. We evaluated the contributions of COPS subunits to HCC tumorigenesis and their utility for prognosis. We comprehensively evaluated the tumor expression pattern and tumorigenic functions of COPS subunits using The Cancer Genome Atlas (TCGA), The Human Protein Atlas and immunohistochemistry. Kaplan–Meier, Cox regression, ROC curve, and nomogram analyses were used to assess the predictive values of COPS subunits for clinical outcome. Expression levels of COPS subunits were significantly upregulated in HCC tissues, which predicted shorter overall survival (OS). Further, Cox regression analysis identified COPS5, COPS7B, and COPS9 as independent prognostic biomarkers for OS. High mutation rates were also found in COPS subunits. Functional network analysis indicated that COPS and neighboring genes regulate ‘protein neddylation’, ‘protein deneddylation’, and ‘protein ubiquitination’. The COPS PPI included strong interactions with p53, CUL1/2/3/4, and JUN. Moreover, the correlations between COPS subunit expression levels and tumor immune cell infiltration rates were examined using TIMER, TISIDB, ssGSEA, and ESTIMATE packages. COPS subunits expression levels were positively correlated with specific tumor immune cell infiltration rates, immunoregulator expression levels, and microsatellite instability in HCC. Finally, knockout of COPS6 and COPS9 in HCC cells reduced while overexpression enhanced proliferation rate and metastasis capacity. Our study revealed that COPS potential biomarker for unfavorable HCC prognosis and indicators of immune infiltration, tumorigenicity, and metastasis.

## INTRODUCTION

Hepatocellular carcinoma (HCC) is one of the most common malignant tumors worldwide. Based on 2020 global cancer statistics, HCC accounts for 8.3% of all cancer deaths [1]. While early-stage HCC patients have a 5-year survival rate of 70% or higher, the 5-year survival rate of patients with advanced liver cancer is less than 5% [2–4] and most patients are diagnosed with intermediate- or advanced-stage disease, resulting in generally poor overall prognosis. One reason for this poor outcome is that patients diagnosed with advanced liver cancer are usually unsuitable for surgery. Recently developed molecularly targeted therapies and immunotherapies are better treatment options for advanced HCC. Nevertheless, the median overall survival is only about one year for patients treated with lenvatinib [5], regorafenib [6] or cabozantinib [7]. Numerous studies on HCC pathogenesis have identified potentially novel therapeutic targets [8, 9], but these findings have not yet translated into substantially prolonged OS. Therefore, the molecular mechanisms underlying HCC require further detailed elucidation. Modern genomics and proteomics techniques utilizing public datasets offer the possibility of identifying aberrantly expressed genes and tumor-related gene networks as prognostic biomarkers and new drug targets.

The ubiquitin–proteasome system (UPS) controls multiple biological functions, including signal transduction, DNA repair, transcriptional regulation, and cell cycle progression, through targeted protein ubiquitination and ensuing degradation. In addition to ubiquitination, several related protein post-translational modification systems also function in eukaryotes such as SUMOylation and NEDDylation. Neddylation is a dynamic and reversible process regulated by deneddylases, of which the COP9 signalosome (COPS) complex is currently the most extensively studied.

The COP9 signalosome is a highly conserved multi-subunit protein complex [10] initially recognized in Arabidopsis as an inhibitor of photomorphogenesis and later found in other unicellular and multicellular eukaryotes [10–13]. The complex is composed of eight subunits (COPS1–COPS8) and the recently discovered ninth subunit CSNAP (COPS9) [14]. The COP9 signalosome shares homology with the 19S “lid” complex of the 26S proteasome, which is thought to function in recognizing ubiquitinated substrates and delivering them to the 20S proteasome core for proteolysis [15]. Thus, it is possible that COPS complexes function to regulate protein degradation. The recognized biological function of COPS is to serve as a deneddylase to remove NEDD8 from neddylated cullin in cullin-RING-E3 ligases (CRLs), which is further enhanced by linking with COPS9 (CSNAP) [16, 17].

Specific COPS subunits are known to influence distinct cellular functions, such as DNA repair, DNA fidelity maintenance, angiogenesis, cell cycle regulation, and microenvironmental homeostasis, which are crucial for carcinogenesis. Indeed, mounting evidence suggests that COPS subunits are associated with the progress and development of cancer. For instance, COPS1 promotes HCC cell metastasis and proliferation by upregulating Cyclin A [18], while COPS2 suppresses degradation of the tumorigenic transcription factor Snail [19]. In contrast to COPS1 and -2, COPS3 inhibits the growth of lung tumors by blocking cell cycle progression [20]. Similar to COPS1 and -2, however, COPS5 promotes tumorigenesis via the degradation of several anti-tumorigenic substrates, including p53 [21], p27 [22], p14ARF [23], Smad4 [24], and the WNT inhibitor DKK177 [25]. Finally, COPS6 assists in the degradation of tumor suppressor p53 via the stabilization of MDM2 [26, 27].

**Figure 1:**
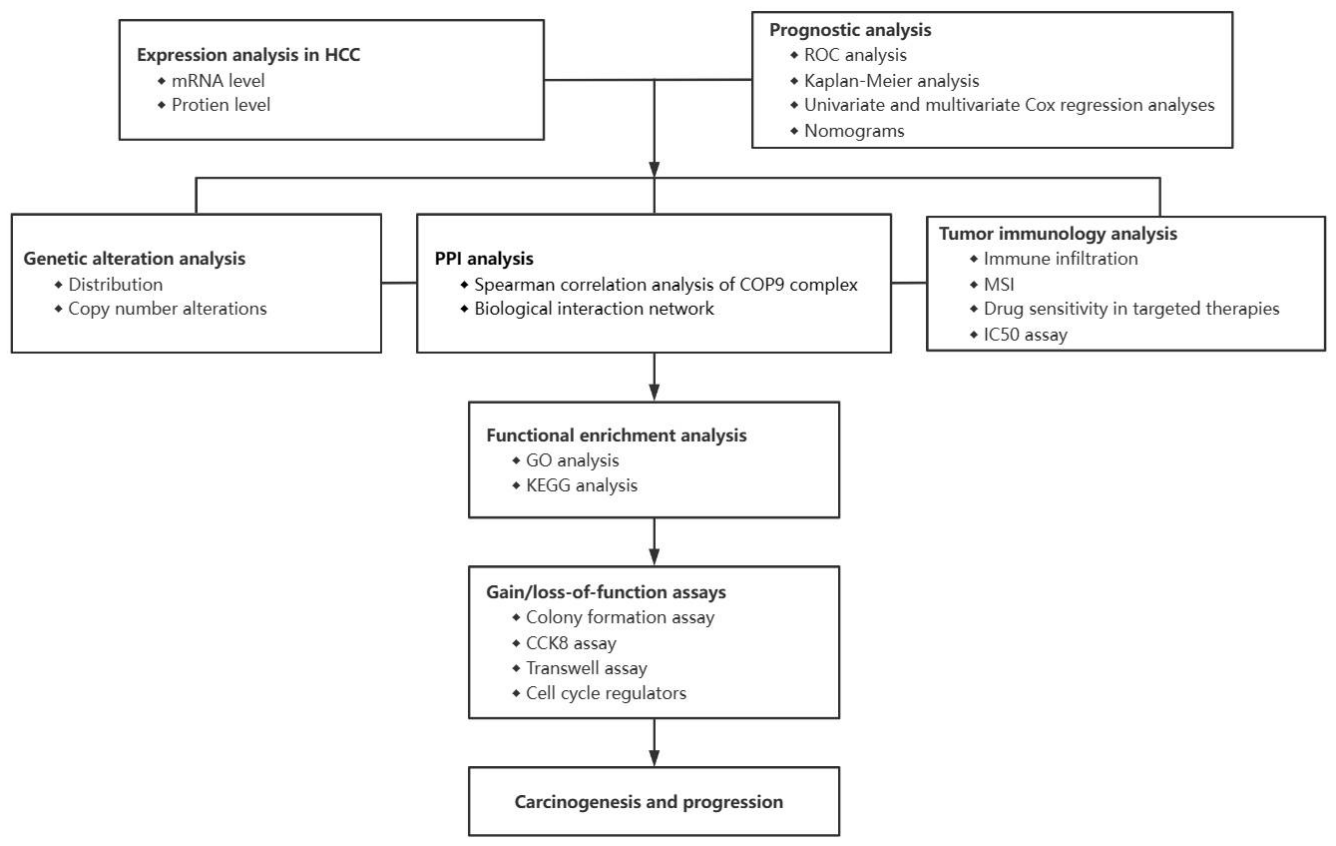
Schematic diagram of the study.

The COP9 signalosome has an emerging role in cancer, although the mechanisms of tumorigenic regulation are still uncharacterized. Therefore, we analyzed the expression levels and predictive values of COPS subunits in HCC by data mining and immunohistochemistry (IHC). We report that the expression levels of COPS subunits were significantly upregulated in HCC compared to normal tissues and that high expression was associated with poor prognosis. We also analyzed the predicted functions and pathways involving COPS and neighboring genes in HCC patients. Furthermore, we found significant correlations between COPS subunit expression levels and tumor immune cell infiltration rates. We suggest that the COPS6 and COPS9 subunits are potential therapeutic targets for HCC treatment. A schematic of this study is shown in Figure 1.

## MATERIALS AND METHODS

### Differential expression analysis of COPS

The UALCAN database (http://ualcan.path.uab.edu) (version 08/16/2022), which contains gene expression profiles and corresponding clinical information from The Cancer Genome Atlas (TCGA), was used to analyze the relationships between COPS subunit expression levels and clinicopathological features. Expression levels were compared between tumor and normal tissues by Student’s t-test, with a p<0.05 (two-tailed) considered statically significant.

The Human Protein Atlas (https://www.proteinatlas.org) (version 23.0), which contains more than 10 million single-cell protein expression profiles generated by immunocytochemistry and immunohistochemistry [28], was utilized to identify differences in protein expression between normal and HCC tissues and to retrieve the related literature. Here, we compared the protein expression levels of COPS subunits based on immunohistochemical images and quantitative proteomics analysis based on mass spectrometry [29–31].

### Prognostic value of COPS complex expression in HCC

The diagnostic utility of COPS subunit expression level was evaluated by ROC curve analysis using the “pROC” R package. Prognostic data were obtained from a published study [32]. Kaplan–Meier, univariate, and multivariate Cox regression analyses were employed for prognosis. Overall survival (OS) was analyzed and plotted using the R “survival” and “survminer” packages, while the R (v3.6.3) package “rms” was used to construct nomograms and calibration plots.

### Genetic alteration analysis

The cBioPortal (https://www.cbioportal.org) (v4.1.18) was used to examine the genomic profiles of COPS subunits in the TCGA and correlations among related genes. The search parameters included somatic mutations, DNA copy number changes (CNAS) from RNA-seq data identified using Genomic Identification of Significant Targets in Cancer (GISTIC), and mRNA expression z-scores generated using RNASeq V2 RSEM.

### COPS-associated PPI network and functional enrichment analysis

The STRING database (http://string-db.org) (version 11.5) was used to construct 10 COPS subunit PPI networks. These networks included physical as well as functional associations, with interaction scores greater than 0.7 considered significant. The DAVID database (https://david.ncifcrf.gov/summary.jsp) (version 2021), which provides functional annotations for large-scale gene and protein lists, was used for function and pathway enrichment analysis of differentially expressed genes.

### Immune infiltration and microsatellite instability analysis

The TIMER database (https://cistrome.shinyapps.io/timer/) [33] was used to assess the associations of COPS subunit expression levels with B cell, CD8+ T cell, CD4+ T cell, macrophage, neutrophil, and dendritic cell infiltration rates. The ssGSEA method from the R package “GSVA” [34] was used to present the infiltration enrichment of 24 common immune cells. Stromal, immune, and ESTIMATE scores of each HCC patient were calculated using the R package “estimate” [35]. Moreover, the TISIDB database (http://cis.hku.hk/TISIDB/index.php) was utilized to analyze the associations between COPS and immunomodulator expression levels in HCC. For microsatellite instability (MSI) analysis, Spearman’s correlation coefficients were calculated between COPS gene expression levels and MSI scores.

### Drug sensitivity analysis

The Gene Set Cancer Analysis (GSCA, http://bioinfo.life.hust.edu.cn/GSCA/#/) (version 09/26/2022), which integrates over 10,000 genomic datasets of 33 cancer types from TCGA and over 750 small molecule drugs from Genomics of Drug Sensitivity in Cancer (GDSC), was used to investigate the correlations between COPS subunit expression levels and drug response.

### Patient samples and IHC

For the analysis of COP9 subunits expression levels, we purchased the human HCC tissue microarray from Shanghai Outdo Biotech company (HLivH028PG01, Shanghai Outdo Biotech, China). All patients were completely informed before the collection of the tissue samples and written informed consent was provided as well. A total of 75 cancer-adjacent normal tissues and cancer tissues from patients with HCC were included.

Tissues were hydrated, antigen repaired and circled. After blocking for 30 min in 10% normal goat serum, anti-MYEOV2 (Novus Biologicals, USA) were applied and incubated overnight at 4° C. After incubation in HRP-secondary antibody, sections were washed with phosphate-buffered saline (PBS) and the chromogen reaction was performed with diamino benzidine (DAB). The Image-Pro Plus 6.0 System, an image analysis system, was used for quantitative analysis.

### Cell culture

The SK-Hep-1 and Hep G2 cell lines were purchased from the Meisen Chinese Tissue Culture Collection and Cell Bank of Shanghai Academy of Chinese Sciences, respectively. Both lines were cultured at 37° C under a 5% CO_2_ atmosphere in DMEM (Gibco, USA) supplemented with 10% fetal bovine serum (Gibco, USA).

### Western blotting

Western blotting was conducted as described [36] using primary antibodies against CyclinB1, CDK4, p18, p21, GAPDH, COPS6 and COPS9. Labeled bands were visualized using horseradish peroxidase (HRP)-linked anti-rabbit or anti-mouse IgGs. All antibodies were purchased from Cell Signaling Technology (USA).

### Cell proliferation analysis

Cell viability was measured using colony formation assay and CCK8 kit (Dojindo, Japan) as described [36]. The inhibitory concentration 50% (IC_50_) of 5-Fluorouracil was determined using a CCK8 assay. After allowing the cells to adhere overnight, complete medium was replaced with medium containing serially diluted 5-Fluorouracil reagent (0, 1, 2, 4, 8, 16, 32, 64, 128, 256 and 512 μM). After incubating for 48 h, 10 μl of CCK8 solution was added to each well for 2 h at 37° C. The optical density of each well at 450 nm was determined using a microplate reader (Thermo Fisher Scientific, USA).

### Transwell assay

Transwell migration and Matrigel invasion assays were conducted to evaluate cell migration and invasion, respectively, using transwell chambers supplied by Corning (USA) as described [36].

### Statistical analysis

SPSS version 26 and GraphPad Prism 8 were utilized for statistical analyses. Results are presented as mean ± standard deviation (SD) of three independent experiments. Group means were compared by Student’s t-test or one-way ANOVA as indicated. Kaplan–Meier curves were constructed to assess OS and group differences were evaluated by log-rank test. A two-tailed P < 0.05 was regarded as statistically significant.

## RESULTS

### Expression of COPS subunits in HCC: associations with clinical pathological parameters

**Figure 2:**
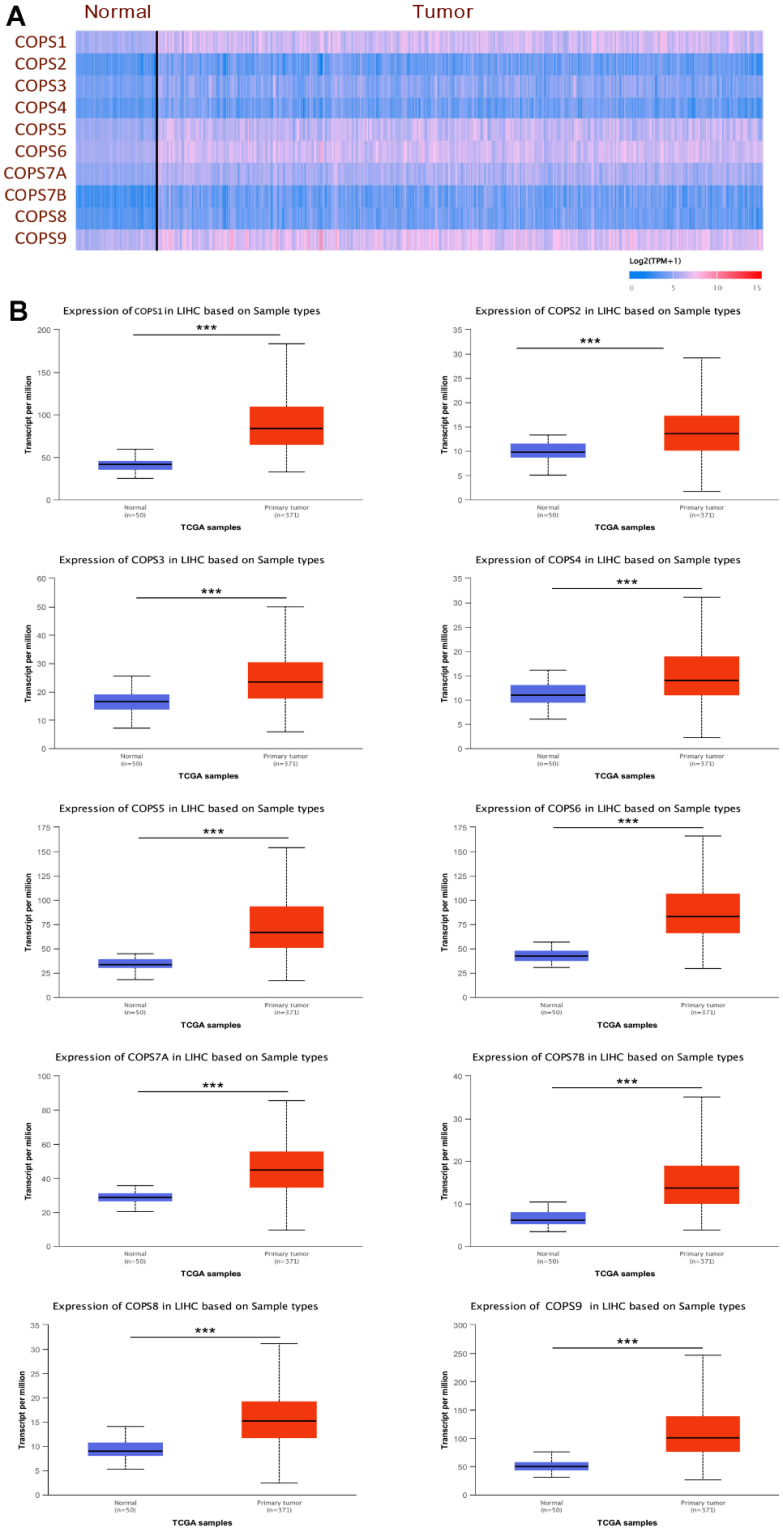
**Elevated expression levels of COPS subunit mRNAs in HCC tissue (from the UALCAN database).** (**A**) Heatmap showing the differential expression levels of COPS subunit mRNAs between HCC and normal liver tissues. (**B**) Direct comparison of COPS subunit mRNAs between HCC and normal liver tissues. ***p < 0.001.

**Figure 3:**
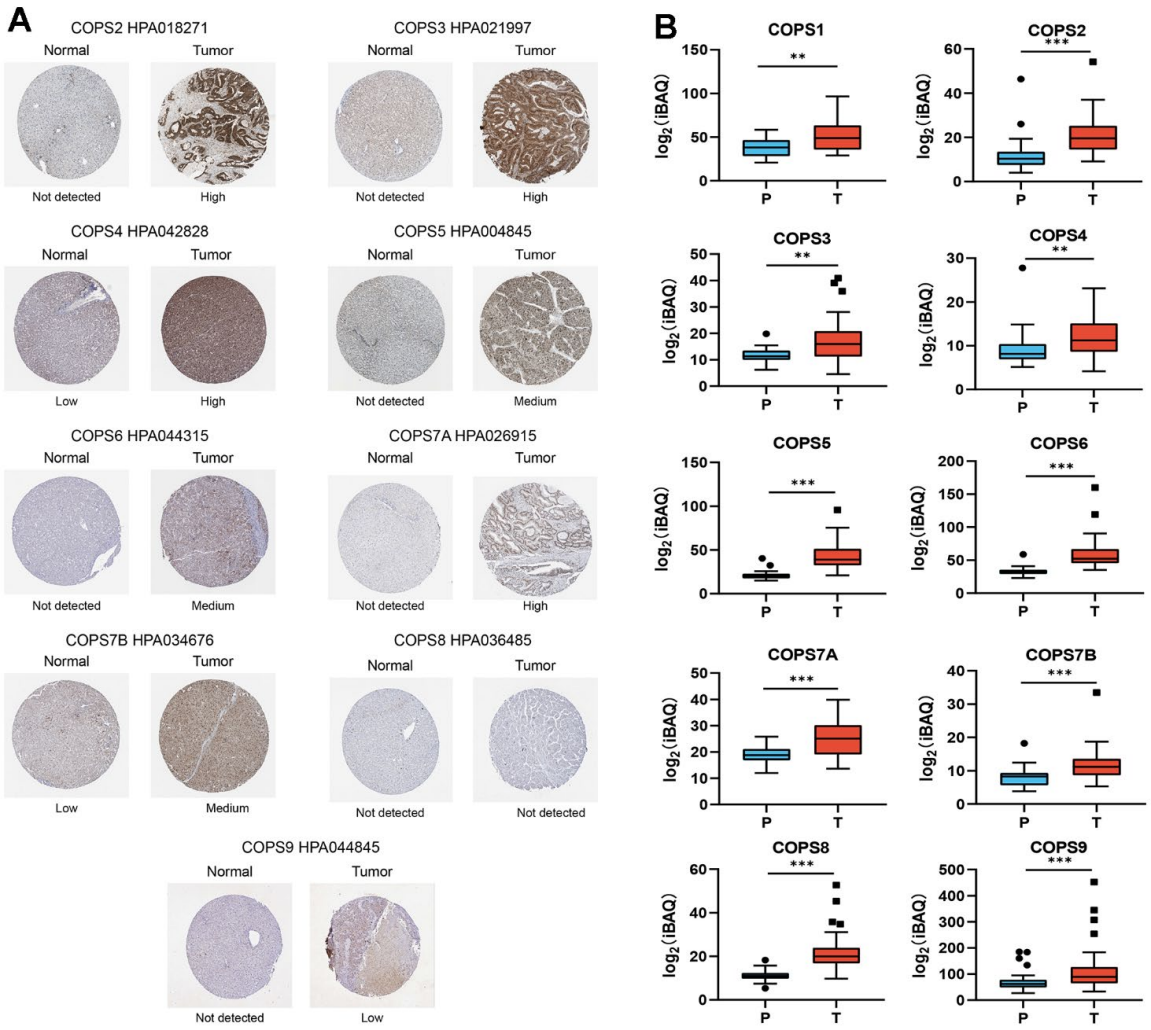
**Elevated expression levels of COPS subunit proteins in HCC tissues as evidenced by immunohistochemistry (from The Human Protein Atlas).** (**A**) Representative images of tissues immunostained with the indicated HPA antibodies. (**B**) Mass spectrometry-based quantitative proteomics analysis of COPS subunit proteins in HCC. **p < 0.01, ***p < 0.001.

We first examined the expression levels of COPS subunits 1, 2, 3, 4, 5, 6, 7A, 7B, 8, and 9 at both mRNA and protein levels using the UALCAN database and The Human Protein Atlas. According to the UALCAN, the mRNA expression levels of all 10 COPS subunits were significantly upregulated in HCC tissues compared to adjacent non-cancerous tissues (Figure 2). Moreover, COPS subunit proteins were differentially expressed in HCC tissues according to immunohistochemistry results included in The Human Protein Atlas (Figure 3A). To further validate the expression levels of COP9 subunits in HCC, we purchased the human HCC tissue microarray from Shanghai outdo biotech company, consisting of 75 pairs of cancer and adjacent normal tissues. We performed IHC staining to detect COPS subunit expression in HCC and adjacent tissues. As expected, the result showed COPS subunit expression was upregulated in HCC tissues (Supplementary Figure 2A–2C). However, considering the small number of samples tested, we further investigated additional proteomics data based on mass spectrometry. These results confirmed variable upregulation of COPS subunit proteins in HCC tissues relative to healthy adjacent tissues (Figure 3B [29], Supplementary Figure 2D [30] and Supplementary Figure 2E [31]).

Analysis of the UALCAN database also revealed that the expression levels of certain COPS subunits were significantly correlated with tumor stage. As shown in Supplementary Figure 1, late-stage HCC patients tended to show higher COPS expression levels. The mRNA expression levels of COPS1, -2, -6, and -9 were higher in late-stage HCC patients (grade 2/3) than grade 1 patients. Further, expression levels of COPS3, -4, -7B, and -8 differed significantly between grade 1 and grade 3 cases, and increased progressively with grade (although expression levels in grade 4 did not show significant variation owing to inadequate case numbers). Thus, COPS subunits are upregulated in HCC tissues at both protein and mRNA levels. Moreover, overexpression is associated with higher tumor grade, suggesting that expression levels may have prognostic value.

### Predictive values of COPS subunits for HCC diagnosis and prognosis

**Figure 4:**
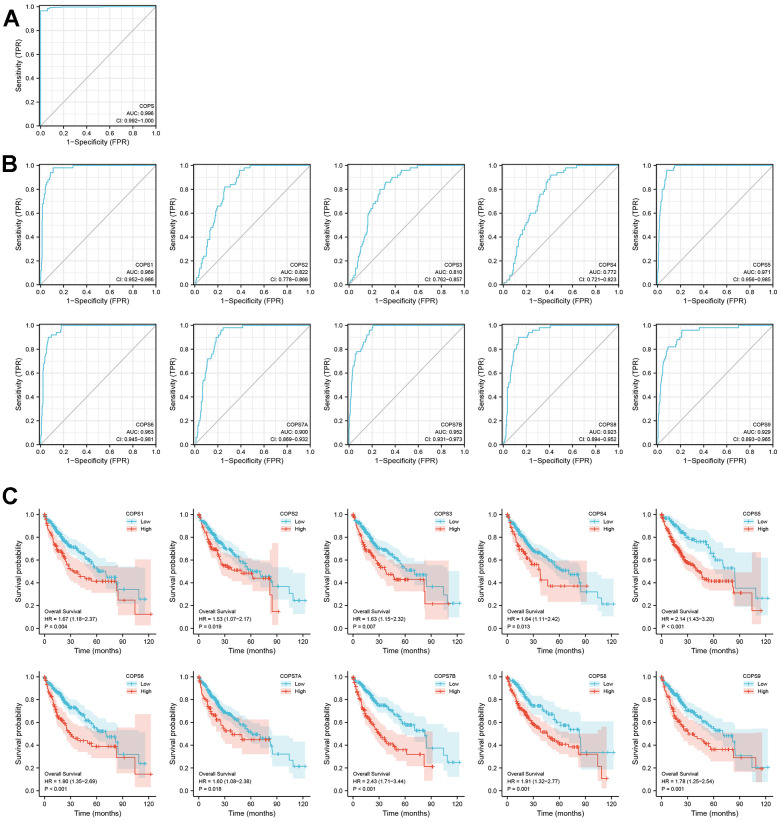
**Prognostic values of COPS subunits for HCC.** ROC analysis of (**A**) COPS complex and (**B**) individual COPS subunits for the diagnosis of HCC. (**C**) Kaplan–Meier survival curves comparing the overall survival (OS) between HCC patients with high or low expression of the indicated COPS subunit (data from the TCGA database).

To assess the clinical values of COPS subunit expression levels for HCC diagnosis, we conducted ROC curve analysis and found that COPS complex expression (any subunit configuration) discriminated HCC from non-HCC with high sensitivity and specificity (area under the curve [AUC]=0.996) (Figure 4A). Moreover, relatively accurate discrimination was also achieved using only expression of COPS1 (AUC=0.969), COPS2 (AUC=0.822), COPS3 (AUC=0.810), COPS4 (AUC=0.772), COPS5 (AUC=0.971), COPS6 (AUC=0.963), COPS7A (AUC=0.900), COPS7B (AUC=0.952), COPS8 (AUC=0.923), or COPS9 (AUC=0.929) (Figure 4B). Kaplan–Meier analysis also indicated that shorter OS was associated with elevated tumoral expression of COPS subunits (Figure 4C).

**Figure 5:**
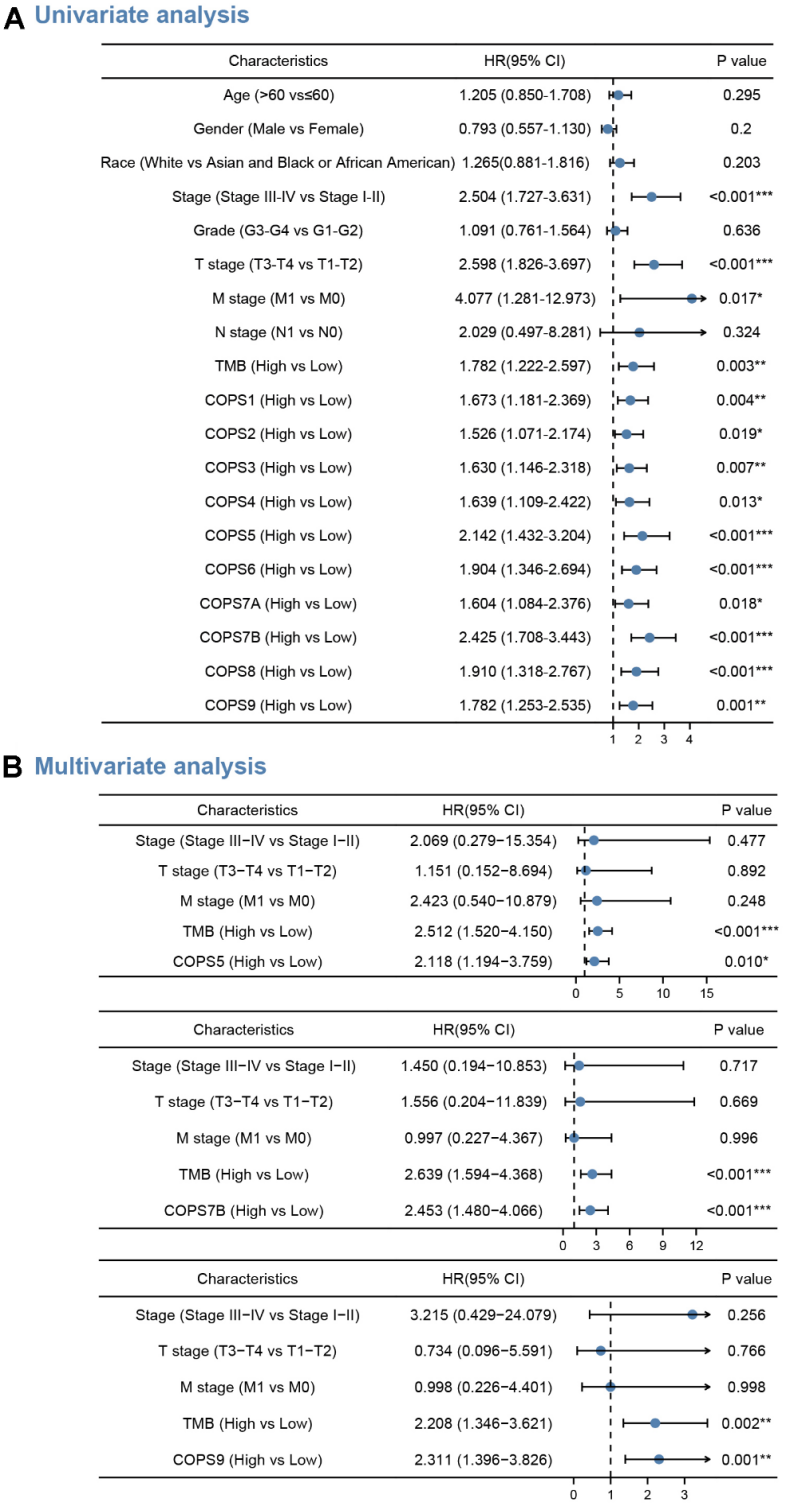
**COPS subunits with prognostic significance.** (**A**, **B**) Forest plots showing the results of univariate analysis (**A**) and multivariate Cox regression analyses (**B**) for the associations between COPS subunit expression levels and OS probabilities. Bars represent the 95% confidence intervals of the hazard ratios. *p < 0.05, **p < 0.01, ***p < 0.001.

In addition, we performed a multivariate Cox proportional hazards regression analysis to further analyze the predictive value of COPS subunits on clinical outcomes (Figure 5). We identified expression levels of COPS5 (HR=2.118, 95%CI: 1.194–3.759, p=0.010), COPS7B (HR=2.453, 95%CI: 1.480–4.066, p<0.0.001), and COPS9 (HR=2.311, 95%CI: 1.396–3.826, p=0.001) as independent predictors of OS (see forest plots in Figure 5A), suggesting high utility as prognostic biomarkers. We also found that Tumor Mutation Burden (TMB), an emerging biomarker to independently predict immunotherapy response, was an independent risk factor for OS (p<0.01). This association is explained by the high mutation loads in tumors, which leads to the formation of new antigens, greater immunogenicity, and improved clinical response to immunotherapy.

**Figure 6:**
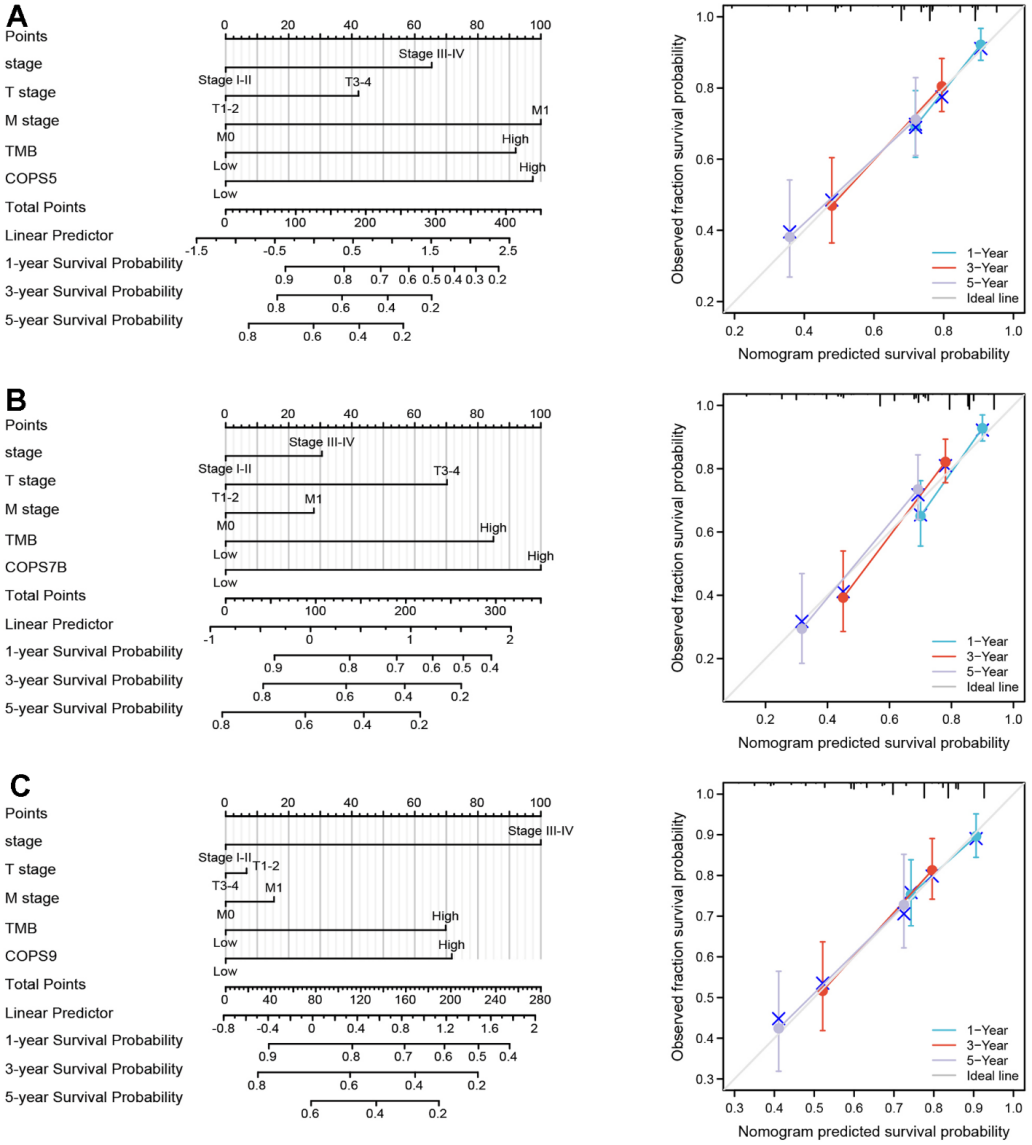
**Construction and validation of nomograms based on COPS subunit expression.** (**A**–**C**) Nomograms constructed to establish the prognostic efficacy of COPS subunits 5 (**A**), 7B (**B**), and 9 (**C**) for predicting 1-, 3-, and 5-year overall survival (OS). Calibration plots are also shown validating the efficiency of nomograms for OS prediction.

All prognostic factors deemed significant by multivariate Cox regression analysis (clinical stage, N stage, M stages, TMB, COPS5, COPS7B, and COPS9) were then used to construct prognostic nomograms for predicting OS, and a calibration curve was drawn to test nomogram efficiency. In the first prognostic model, clinical M stage was the strongest contributor to 1-, 3- and 5-year OS, followed closely by COPS5 expression. In the second prognostic model, COPS7B expression was the strongest contributor to 1-, 3- and 5-year OS, followed closely by TMB. In the third and fourth models, clinical stage was the strongest contributor to 1-, 3- and 5-year OS, followed closely by COPS9 expression. These prognostic nomograms yielded C-index values of 0.689, 0.701, and 0.679. Moreover, the calibration curves showed that nomogram-predicted probabilities were close to the ideal reference line for 1-, 3- and 5-year OS (Figure 6). Thus, these user-friendly graphical tools allowed us to easily determine the one-, three- and five-year OS probabilities for each HCC patient.

### Genetic mutations of COPS subunits in HCC

**Figure 7:**
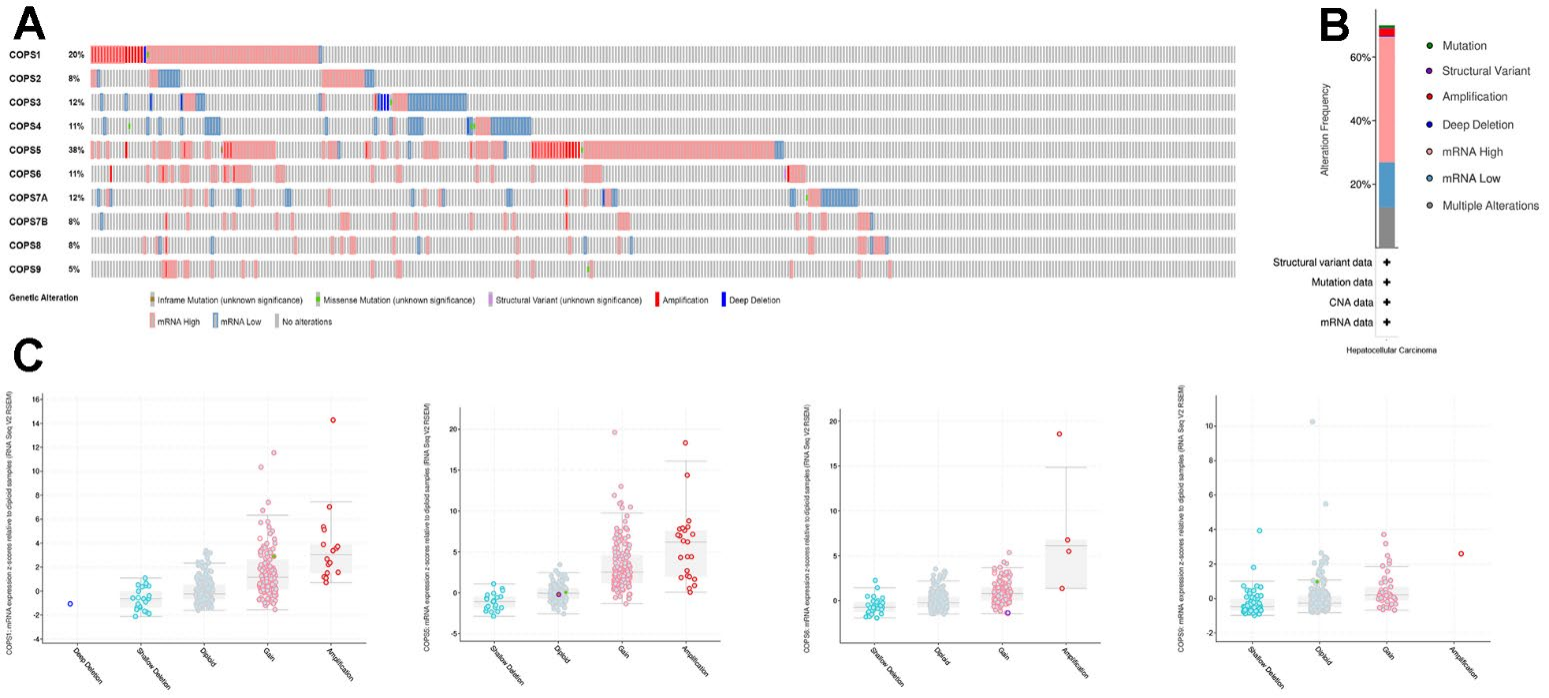
**Alterations of COPS subunit genes (from cBioPortal).** (**A**) Summary of COPS subunit gene mutation rates. (**B**) Genetic alteration summary of the COPS complex in HCC. (**C**) COPS subunit gene mutation types.

Next, cBioPortal was utilized to evaluate COPS subunit gene mutations and copy number alterations in HCC. Seventy percent (260/372) of HCC patients in the database carried COPS subunit gene alterations, with highest rates in COPS5 (38%), COPS1 (20%), and COPS3/7A (12%) (Figure 7A, 7B). Upregulation of mRNA expression was the most common COPS subunit gene alteration in HCC patients. Moreover, significant correlations between mRNA expression levels and copy numbers were observed for COPS1, COPS5, COPS6, and COPS9, suggesting that overexpression resulted from gene duplication (Figure 7C).

### COPS subunit gene network construction and functional enrichment analysis

**Figure 8:**
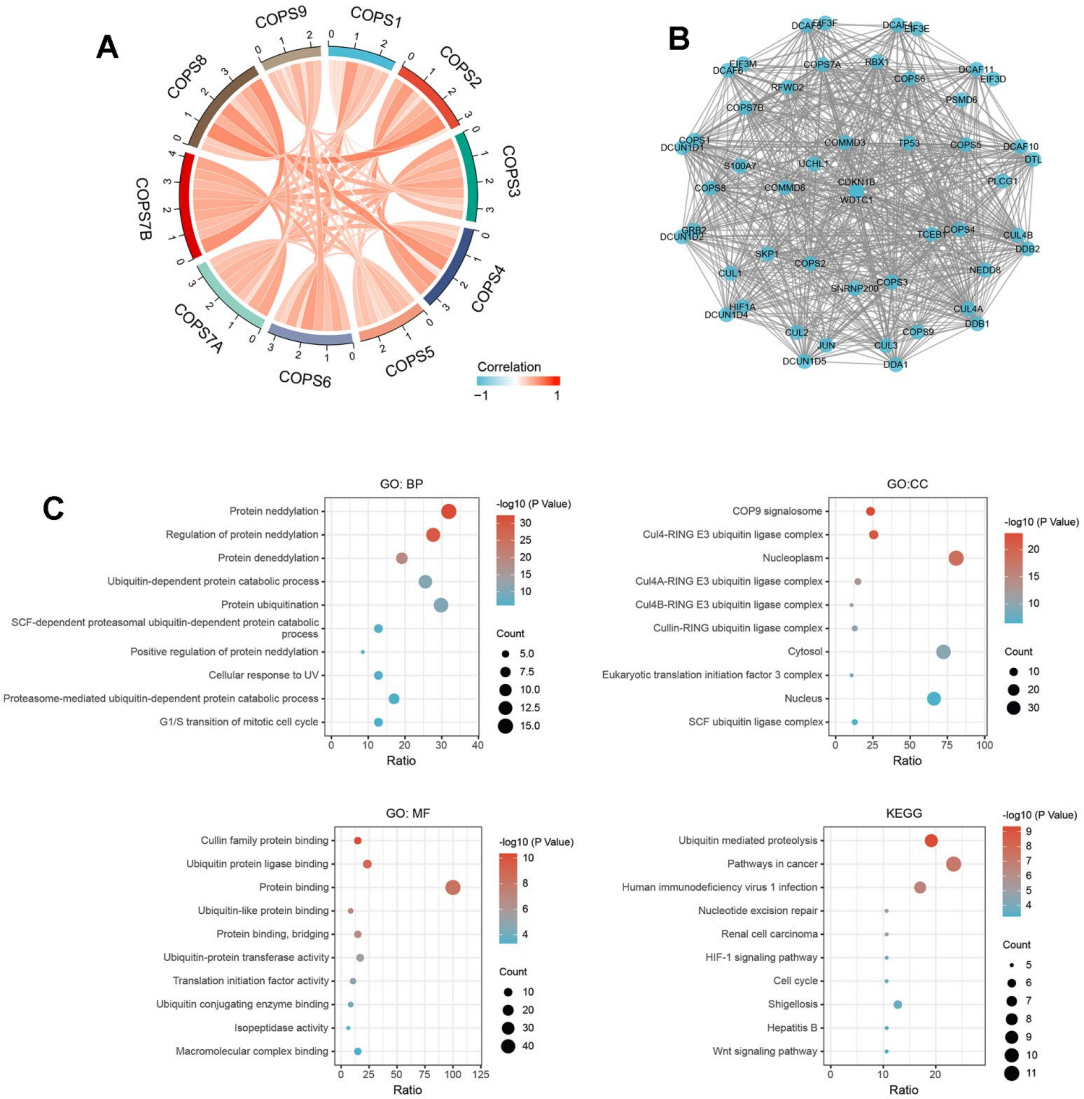
**Functional enrichment analyses of COPS subunit genes and neighboring genes in HCC (from STRING and DAVID).** (**A**) Associations among COPS complex components in HCC revealed by Spearman correlation analysis. (**B**) Biological interaction network of COPS subunit genes and neighboring genes. (**C**) GO and KEGG enrichment analysis showing biological processes, cellular components, molecular functions, and molecular pathways of COPS subunits and neighboring network genes.

We then examined the correlations among expression levels of the ten COPS subunits using the TCGA database, and a positive correlation was found between each individual subunit component (see chord diagram in Figure 8A). To predict the functions of these COPS subunits in humans, a PPI network was constructed with the 40 nearest-neighbor genes using STRING. The final network included 60 nodes, 926 edges, and particularly strong interactions with TP53, CUL1/2/3/4, and JUN (Figure 8B). According to GO enrichment analysis (Figure 8C), COPS subunit genes and 40 nearest-neighbor genes are involved predominantly in ‘protein neddylation’, ‘regulation of protein neddylation’, ‘protein deneddylation’, ‘ubiquitin-dependent protein catabolic process’, and ‘protein ubiquitination’. The proteins encoded by these genes are located primarily in ‘COP9 signalosome’, ‘Cul4-RING E3 ubiquitin ligase complex’, ‘nucleoplasm’, ‘Cul4A-RING E3 ubiquitin ligase complex’, and ‘Cul4B-RING E3 ubiquitin ligase complex’, and are enriched in the molecular function annotations ‘cullin family protein binding’, ‘ubiquitin protein ligase binding’, and ‘protein binding’. According to KEGG enrichment analysis, these genes are involved in ‘Ubiquitin mediated proteolysis’, ‘Pathways in cancer’, ‘Nucleotide excision repair’, ‘HIF-1 signaling pathway’, ‘Cell cycle’, and ‘Wnt signaling pathway’ (Figure 8C).

### COPS subunit expression levels were associated with HCC tumor immune cell infiltration

**Figure 9:**
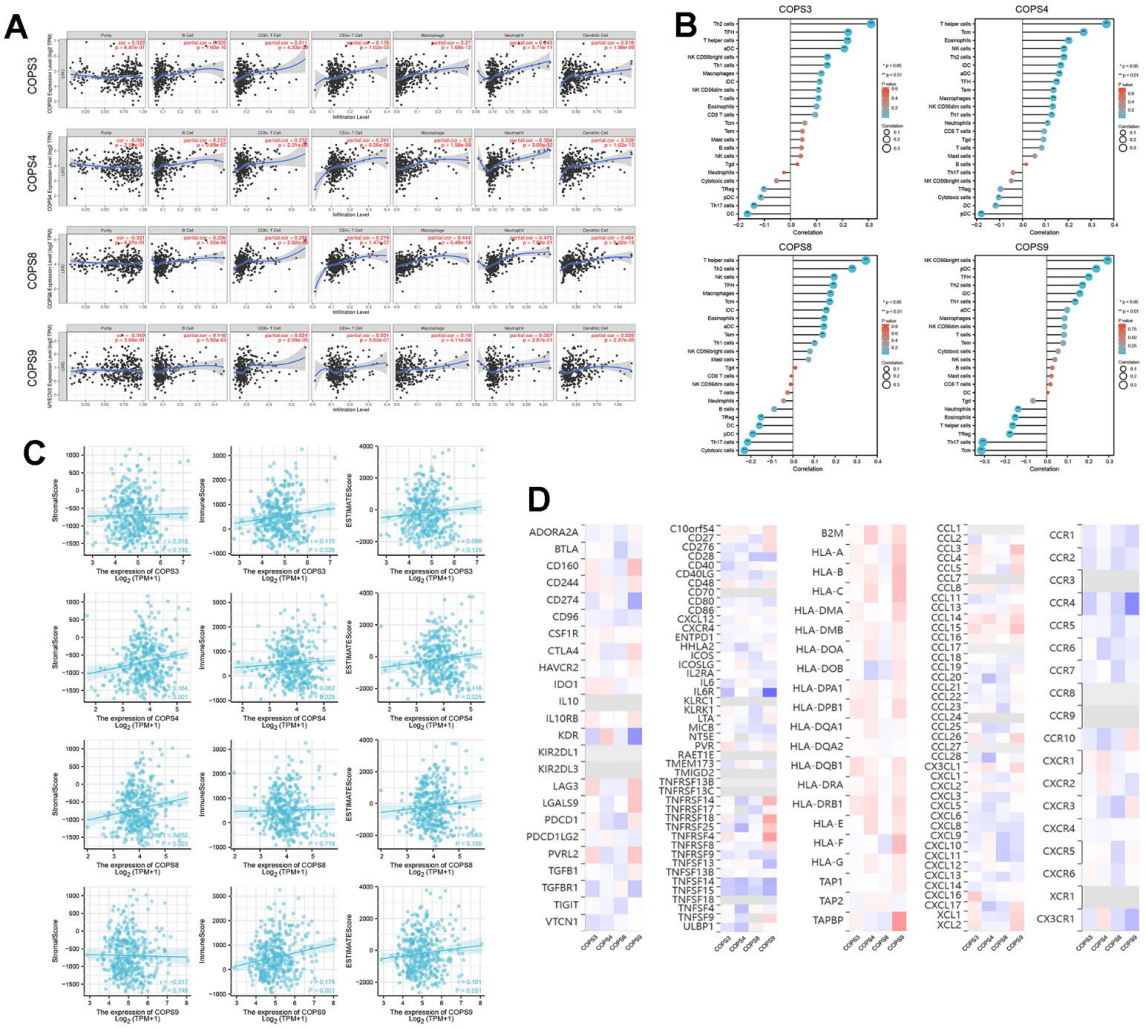
**Correlations between COPS subunit gene expression levels and immune cell infiltration rates.** (**A**) Correlations of COPS subunit expression levels with infiltration rates of B cells, CD8+ T cells, CD4+ T cells, macrophages, neutrophils, and dendritic cells in HCC (from the TIMER2.0 database). (**B**) Relationships among the infiltration levels of 24 immune cell types and COPS subunit expression profiles (based on the ssGSEA R package). (**C**) Relationships between COPS subunit expression levels and stromal score, immune score, and ESTIMATE score. (**D**) Heatmaps showing the correlations between COPS subunit expression levels and immunostimulators, immunoinhibitors, MHC molecules, chemokines, and chemokine receptors in HCC (TISIDB).

Mounting evidence suggests that tumor-infiltrating immune cells regulate tumor development and progression [37], but it is unclear whether elevated expression of COPS subunit genes affects immune cell recruitment in HCC. Analysis using the TIMER database revealed that COPS3, COPS4, COPS8, and COPS9 expression levels were positively correlated with the numbers of infiltrating B cells, CD8+ T cells, CD4+ T cells, macrophages, neutrophils, and dendritic cells (Figure 9A). Application of the “GSVA” and “estimate” R packages further revealed that COPS3, COPS4, COPS8, and COPS9 expression levels were positively correlated with the infiltration rates of most immune cells (Figure 9B). We also found that elevated expression levels of COPS4 and COPS8 were associated with higher stromal score, an index of immune infiltration. Moreover, the expression levels of COPS3 and COPS9 were positively correlated with immune score and COPS4 expression level with ESTIMATE score (Figure 9C). Also, COPS subunit expression levels were associated with immunostimulators, immunoinhibitors, MHC molecules, chemokines, and chemokine receptors of infiltrating immune cells in HCC (Figure 9D). These results thus identify additional prognostic biomarkers and therapeutic targets for HCC.

**Figure 10:**
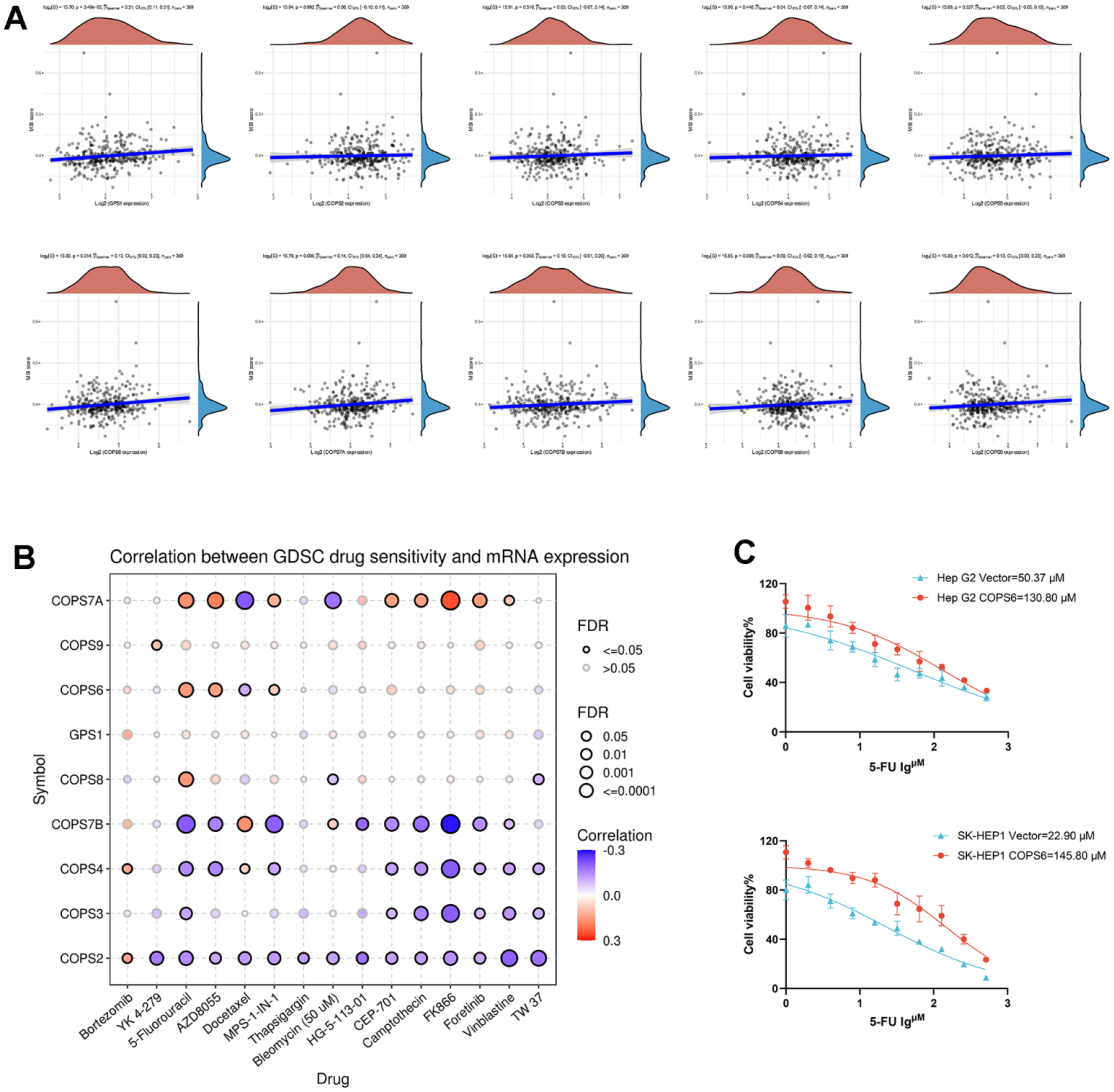
**Microsatellite stability (MSI) and drug-sensitivity analysis of COPS subunits in HCC.** (**A**) Correlations between COPS subunit expression levels and MSI in HCC. (**B**) Correlations between COPS subunit expression levels and GDSC drug sensitivity in pan-cancer. (**C**) The effect of COPS6 overexpression on IC50 value of 5-Fluorouracil in Hep G2 and SK-HEP1cells.

The microsatellite stability index has been reported to predict immunotherapy response [38], and the results presented here revealed that COPS subunit expression levels are predictive of tumor immune cell infiltration. To examine if COPS subunits can also serve as biomarkers for drug screening, we analyzed the correlations with MSI and found positive correlations with the expression of COPS1 (p=3.49e^-5^), COPS6 (p=0.014), COPS7A (p=0.006), and COPS9 (p=0.012) (Figure 10A), while there were no significant correlations between MSI and expression levels of COPS2, -3, -4, -5, -7B, and -8 (Figure 10A). In addition, we examined the relationships between COPS subunits levels and sensitivities to various drugs using the GSCA database and found COPS subunit expression levels were negatively correlated with some or most drugs in the GDSC database (Figure 10B). We further validated the correlation between 5-Fluorouracil sensitivity and COPS6 expression level in HCC. As shown in Figure 10C, IC50 value for 5-Fluorouracil increased in COPS6-overexpressing Hep G2 and SK-HEP1 cells, which is consistent with the predicted results of the GSCA database (Figure 10B). This has reference significance for conducting clinical research studies and guiding the clinical medication of HCC treatment.

### COPS6 and COPS9 are essential for cell proliferation and metastasis of HCC cells

**Figure 11:**
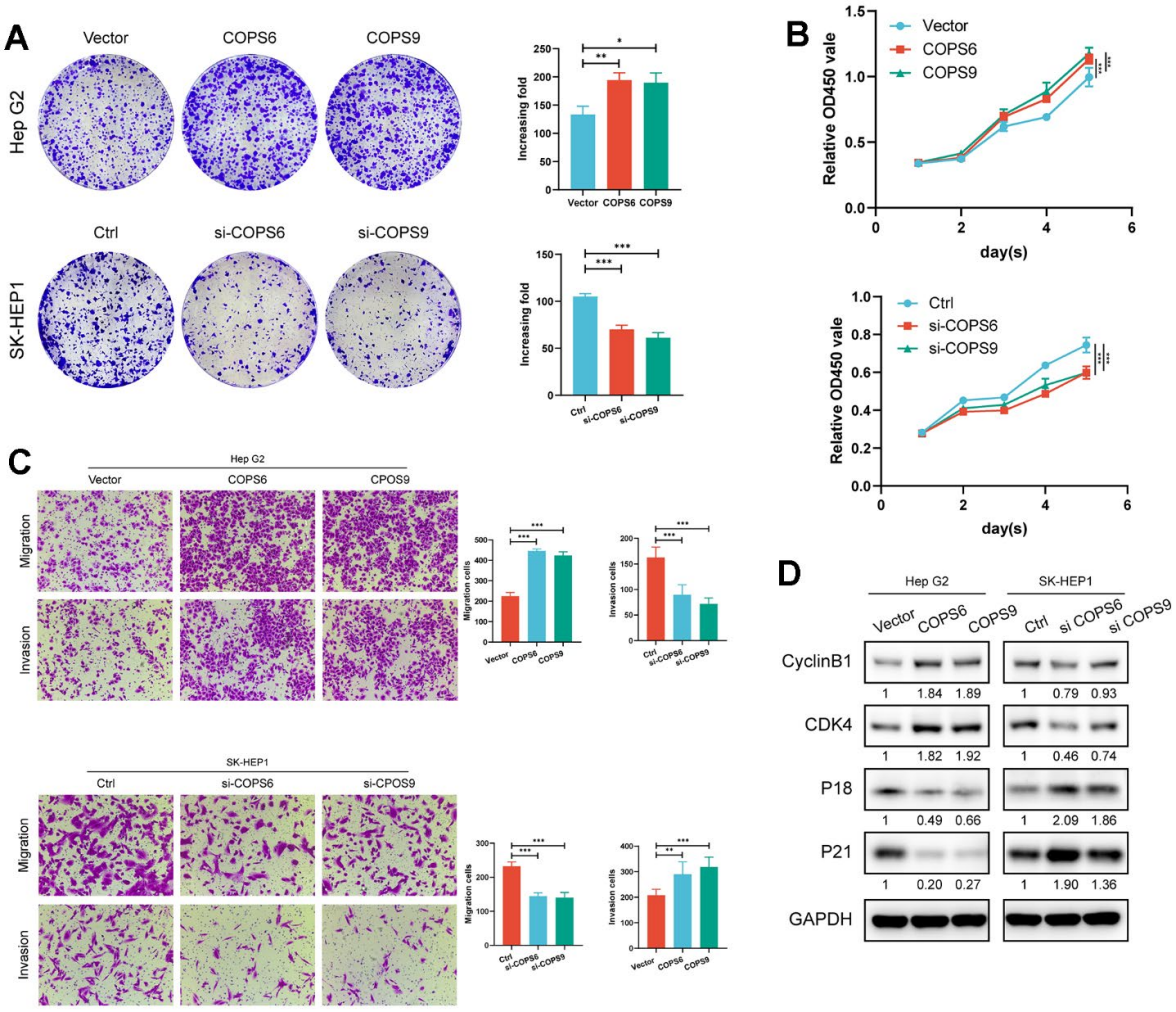
**Role of COPS6 and COPS9 in the proliferation, migration, invasion, and cell cycle progression regulation of HCC cells.** (**A**) Colony formation assays showing the role of COPS6 and COPS9 on HepG2 and SK-HEP-1 cell proliferation. (**B**) Cell viability of HepG2 and SK-HEP-1 cell lines were determined by CCK-8 assays. (**C**) The effects of COPS6 and COPS9 on cell migration and invasion were determined by transwell assays in Hep G2 and SK-HEP-1 cell lines. (**D**) The relative expression levels of cyclin B1, CDK4, p18, and p21 were examined by Western blotting in Hep G2 and SK-HEP-1 cells. *p < 0.05, **p < 0.01, ***p < 0.001.

To examine the biological function of COPS6 and COPS9 in HCC, Hep G2 and SK-HEP-1 cells were transfected separately with COPS6 and COPS9 overexpression or knockdown vectors (Supplementary Figure 3). Both COPS6 knockdown and COPS9 knockdown significantly suppressed while overexpression significantly increased colony formation (Figure 11A). Both COPS6 and COPS9 knockdown also reduced viable HCC cell number after several days in culture as estimated by CCK8 assay, while overexpression significantly increased (Figure 11B), suggesting that COPS6 and COPS9 accelerate HCC tumor growth. Both COPS6 and COPS9 overexpression promoted HCC cell migration and Matrigel invasion in transwell assays, while COPS6 siRNA transfection or COPS9 siRNA transfection repressed transwell migration and Matrigel invasion (Figure 11C). Further, COPS6 or COPS9 overexpression enhanced the expression levels of cell cycle regulators CyclinB1 and CDK4, but downregulated the expression of cyclin-dependent kinase inhibitors p18 and p21 according to western blot analysis (Figure 11D). Collectively, these results indicate that COPS6 and COPS9 regulate the tumorigenicity and metastasis of HCC.

## DISCUSSION

The COP9 signalosome is a highly conserved multi-subunit complex localized mainly in the nucleus of eukaryotic cells. The individual subunits of COPS are highly homologous to the lid complex (19S) of the 26S proteasome, suggesting functions in ubiquitin- and proteasome-dependent protein degradation. Tumor cells carry a high unfolded protein burden due to mutations in protein-coding sequences and concomitant abnormal ubiquitination [39]. Thus, aberrant expression of COPS subunits may contribute to cancer development and progression. Indeed, COPS subunit overexpression has been detected in breast cancer [40–42], HCC [18, 43], colorectal cancer [44], gastric cancer [23, 45], pancreatic cancer [46, 47], myeloma [48], non-small cell lung cancer [49], prostate cancer [50], and osteosarcoma [51]. Overexpression of COPS5 has also been implicated in lymph node metastasis [52] and histological tumor progression [53]. In addition, high expression levels of COPS5 and COPS6 were found to predict poorer prognosis in cancer patients [44, 47, 54, 55]. Thus, COPS subunit genes may be valuable biomarkers and therapeutic targets for HCC.

COPS subunits have been reported to play a dual role in tumor development. For instance, COPS2 is considered a putative tumor suppressor gene. Carvalho and colleagues identified COPS2 as a possible candidate target gene for miR-15a-3p to inhibit the progression of colorectal adenoma [56]. Alternatively, COPS3 may promote clear cell renal cell carcinoma progression by regulating phospho-AKT (Thr308), Cyclin D1, and Caspase-3 expression [57], while COPS5, -6, and -8 overexpression enhanced cellular proliferation [23, 45, 46, 58, 59], EMT [42, 55, 60, 61], and vascular invasion [62]. Zheng and colleagues also found that KRT19P3 suppressed gastric tumor growth and metastasis through a COPS7A-regulated NF-κB pathway, suggesting that COPS7A acts as a suppressor of gastric cancer [63]. COPS9 is a newly discovered subunit of COP9 with limited research currently, and it has potential research significance. The lack of COPS9 yields a cellular phenotype characterized by reduced cell proliferation and a flattened and enlarged morphology [14]. COPS functions as a molecular platform for proteolysis and signal transduction, and is involved in regulating multiple intracellular signal transduction pathways promoting the occurrence and development of tumors. To assess the functions of COPS subunits and associated regulatory networks in HCC, we conducted a series of bioinformatics analyses and experiments on cultured HCC cells overexpressing or underexpressing COPS subunits.

We first found that the expression levels of multiple COPS subunits were upregulated at both mRNA and protein levels in HCC tissue samples compared to normal liver tissue samples according to archived results in the UALCAN database, Human Protein Atlas and IHC assay. The mRNA expression levels of COPS were also associated with clinicopathologic parameters, and both Kaplan–Meier and Cox regression analyses further identified elevated expression levels of specific COPS subunits as independent risk factors for shorter OS. We also found several alterations in COPS subunit genes, including mutations and gene duplications. Therefore, COPS subunit expression levels may be useful for HCC diagnosis and prognosis, a notion that merits further clinical verification.

To further analyze the tumorigenic functions of COPS subunits, we constructed PPI networks with neighboring genes and found that TP53, CUL1/2/3/4, and JUN had higher combined scores with COPS. Further, these proteins all shared the GO biological process ‘protein neddylation’ and the KEGG pathway ‘Ubiquitin mediated proteolysis’. Consistent with these *in silico* results, the COP9 signalosome serves two principal functions in eukaryotic cells, deneddylation and phosphorylation. The cycling between neddylation and deneddylation is a dynamic process effectively regulated by COPS. The 19S lid complex of the proteasome functions in recognizing and funneling ubiquitinated substrates to the proteolytic core complex for degradation. However, the COP9 signalosome is highly structurally and functionally homologous with the 19S lid complex. Further, COPS5 is at the enzymatic core of COPS and functions to remove NEDD8 from cullin proteins. Similarly, the lid sub-complex subunit RPN11 removes ubiquitin from proteasome substrates. Therefore, the COPS complex may substitute for the lid complex to coordinate the activity of SCF E3 ubiquitin ligases and the 26S proteasome in proteolysis [15]. In fact, COPS promoted the cleavage of the NEDD8–CUL1 conjugate (deneddylation), which in turn inhibited the E3 ubiquitin ligase activity of CRLs [64]. In the absence of COPS, the E3 ubiquitin ligase SCF^TIR1^ had a significantly reduced ability to degrade its substrates [65]. Therefore, cullin-based E3 ubiquitin ligase–mediated responses are likely regulated by COPS. The COP9complex is also involved in the phosphorylation of p53/TP53, c-Jun/JUN, and I-kappa-B-alpha/NFKBIA. COPS-specific phosphorylation targets intracellular p53 for ubiquitin-26S proteasome-dependent degradation by interacting with the N-terminus of p53 [66]. COPS also phosphorylated c-Jun at Ser63 and Ser73 of the N-terminal transactivation domain, which prevented degradation by the ubiquitin-like system [67]. These results suggest that COPS complexes may suppress or promote cancer-related signaling pathways by regulating protein degradation.

Infiltrating immune cells in the tumor microenvironment have been shown to regulate tumor development and metastasis, and thereby to critically impact patient prognosis [37]. Currently, immunotherapy has been reported to be effective for cancer patients, including HCC [68]. Therefore, we examined if COPS subunit expression is associated with altered expression of immune biomarkers and potential immunotherapeutic targets. Indeed, elevated COPS subunit expression was associated with greater immune cell infiltration. Meanwhile, we revealed positive correlations between COPS1, -6, -7A, and -9 expression and MSI in HCC, implying that patients with elevated COPS expression may be more responsive to immunotherapy. Activation of PD-1/PD-L1 signaling allows tumors to evade antigen-specific T-cell immunologic responses. However, studies have indicated a close relationship between COPS and PD-L1. COPS5 and COPS6 can prevent PD-L1 from degrading, thereby maintaining PD-L1 stability in cancer cells [69, 70]. In addition, inhibition of COPS5 by curcumin reduced the expression of PD-L1 and sensitized cancer cells to anti-CTLA4 treatment. Therefore, the relationship between COPS and PD-L1 provides important clues to the regulatory mechanisms of immune evasion, suggests that COPS may modulate anti-tumor immunity and sensitivity to immunotherapy, and identifies COPS subunits a potential biomarker of immune infiltration in HCC.

Finally, we conducted a series of gain- and loss-of-function assays *in vitro* to verify the functions of COPS6 and COPS9 in HCC. Overexpression of COPS6 or COPS9 increased HCC cell proliferation, migration, and invasion while knockdown suppressed these pro-tumorigenic and metastatic properties. In addition, we observed increased p18 and p21 expression levels after COPS6 or COPS9 overexpression and substantial decreases in p18 and p21 expression levels after COPS6 or COPS9 knockdown. It has been demonstrated that COPS-mediated regulation of cell cycle regulators is essential for a variety of tumor types. For instance, COPS5 accelerated the degradation of p27 and overcame p27-mediated cell cycle arrest in the G1 phase [71]. Mechanistically, COPS5/COPS6 induced the cytoplasmic translocation and subsequent degradation of p27 [71, 72]. In nasopharyngeal cancer cells, Jab1 and p27 were shown to interact directly, with Jab1 facilitating proteasome-dependent p27 degradation [22]. COPS subunits also regulate the expression of other cell cycle-associated genes. COPS3 knockdown induced cell cycle arrest at G0/G1 phase by upregulating p21 and downregulating CDK4 and cyclin B1 in lung adenocarcinoma cells [20], while COPS5 expression facilitated MDM2-mediated p53 ubiquitination, nuclear export, and degradation [73]. Similarly, COPS6 facilitated cancer cell growth through p53 ubiquitination and degradation [26, 27]. Here, we suggested that COPS6 and COPS9 were able to downregulate the expression of p18 and p21. Therefore, aberrant overexpression of COPS6/COPS9 in HCC cells facilitated cancer cell growth, which may be attributed to the ubiquitination and degradation of p18 and p21.

These analyses identified several COPS subunits as independent predictive biomarkers of HCC clinical outcome and revealed several potential mechanisms by which COPS subunits may promote HCC tumorigenesis, including aberrant protein degradation and promotion of tumor infiltration by immune cells. Nonetheless, this study has several limitations. First, most of these findings were derived from bioinformatics analyses of public databases and thus require verification by *in vivo* experiments and clinical trials. Second, the pathogenic mechanisms underlying COPS subunit-mediated tumorigenesis were not investigated. We demonstrated that COPS6 and COPS9 promote cell cycle progression, but it is unknown whether these effects are dependent on p21 and p18 degradation via a ubiquitin-mediated process. Future studies should focus on COPS subunits such as COPS6 and COPS9 as potential targets for HCC therapy.

These findings suggest that the COP9 signalosome is a potential biomarker for HCC diagnosis and prognosis as well as a promising treatment target for preventing HCC progression and metastasis. We propose that this data mining and bioinformatics strategy can provide clues to HCC pathogenesis and potentially effective treatment targets.

## Supplementary Material

Supplementary Figures
