# Supplementary material for: COP9 signalosome complex is a prognostic biomarker and corresponds with immune infiltration in hepatocellular carcinoma
Source: Aging (Albany NY). 2024 Mar 11;16(6):5264–87. doi: 10.18632/aging.205646 (PMC11006475; doi:10.18632/aging.205646)
Supplement: Supplementary Figures [file aging-16-205646-s001.pdf]

SUPPLEMENTARY FIGURES

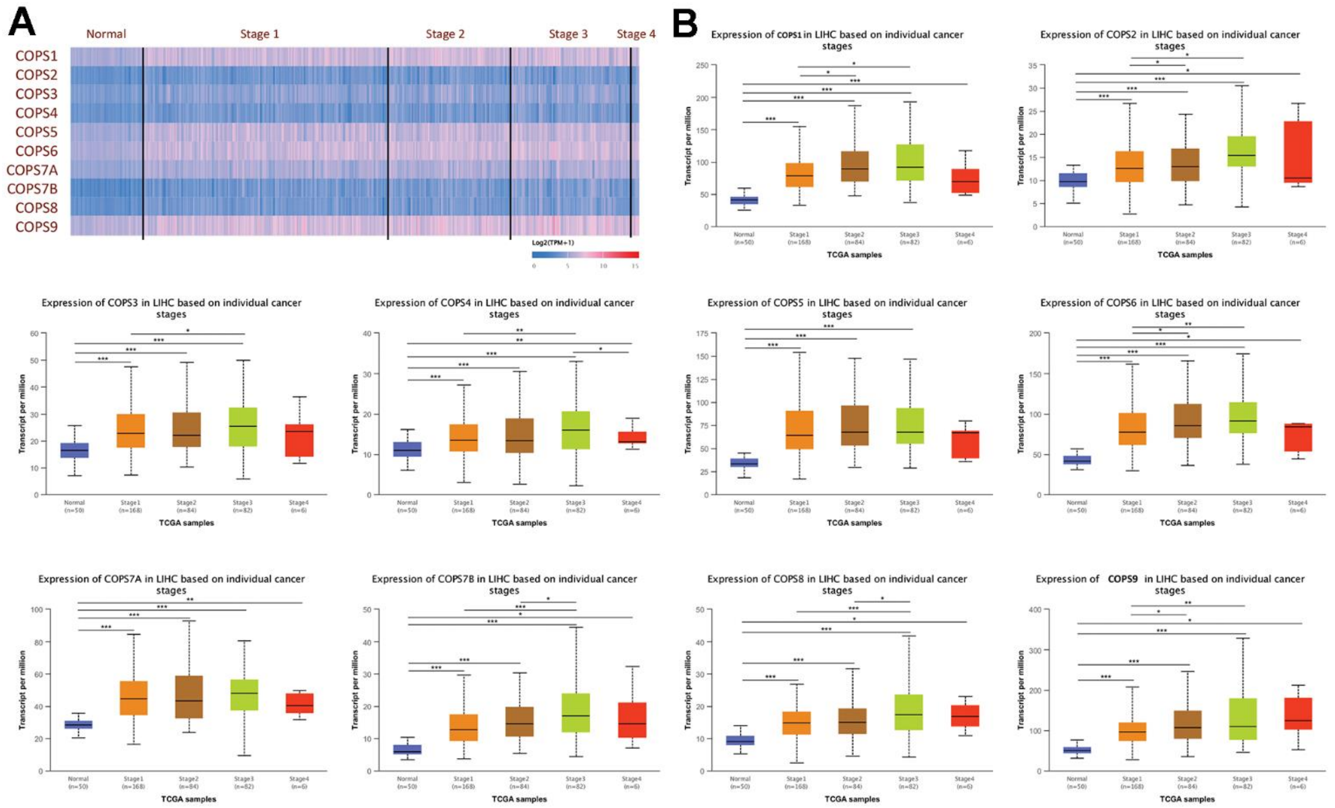

Supplementary Figure 1. Greater COPS subunit mRNA expression levels are associated with higher HCC tumor stage (from UALCAN). (**A**) Heatmap showing the differential expression of COPS subunits in HCC patients with different tumor grades. (**B**) Expression levels of COPS subunits in HCC patients with different tumor grades. *p < 0.05, **p < 0.01, ***p < 0.001.

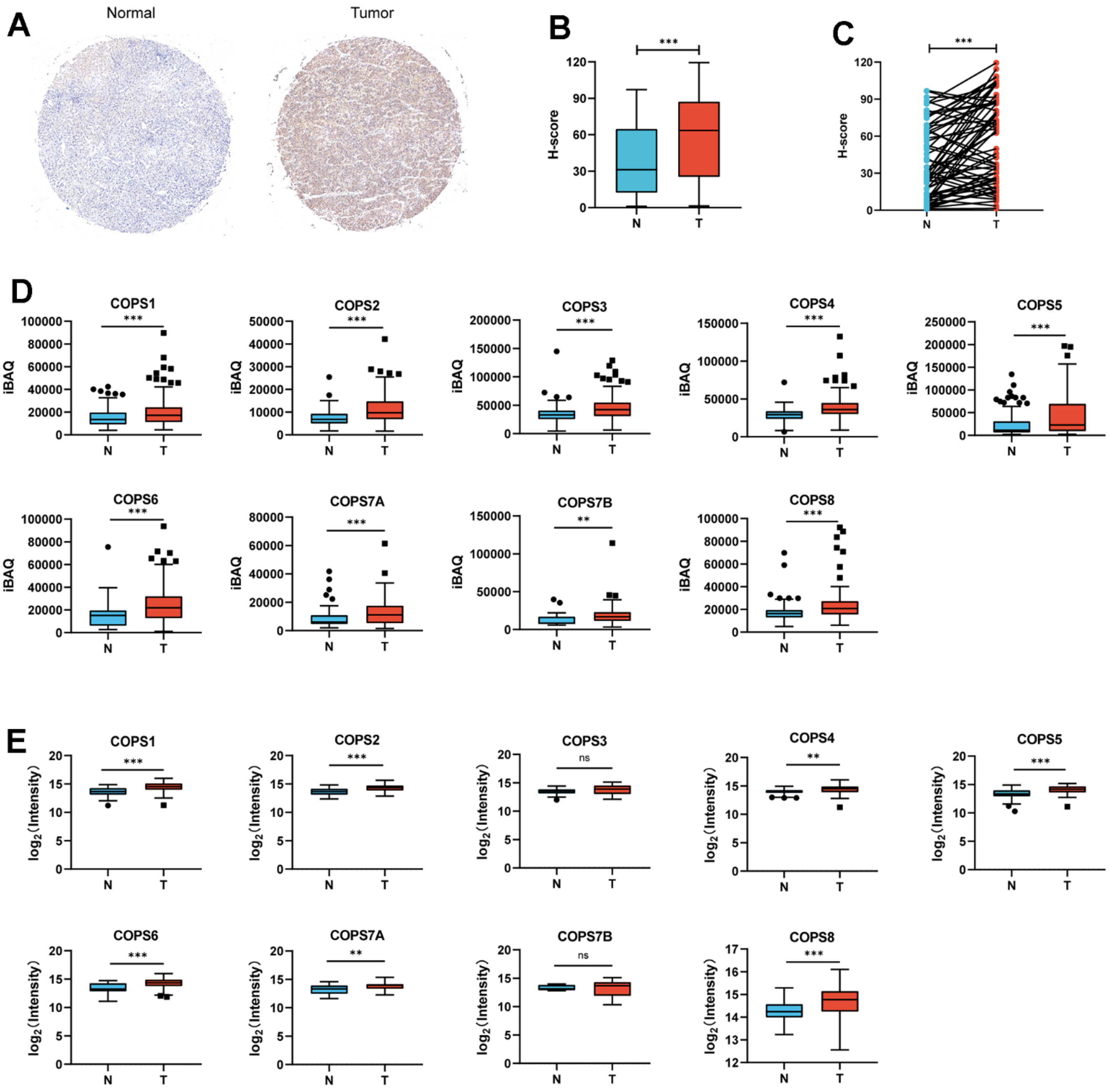

Supplementary Figure 2. COPS subunits were upregulated in HCC. (**A**, **B**) IHC analysis of COPS9 protein levels in adjacent tissues and HCC tissues. (**C**) COPS9 expression in paired adjacent tissues and HCC tissue samples. (**D**, **E**) Mass spectrometry-based quantitative proteomics analysis of COPS subunit proteins in HCC. **p < 0.01, ***p < 0.001. ns, no significance.

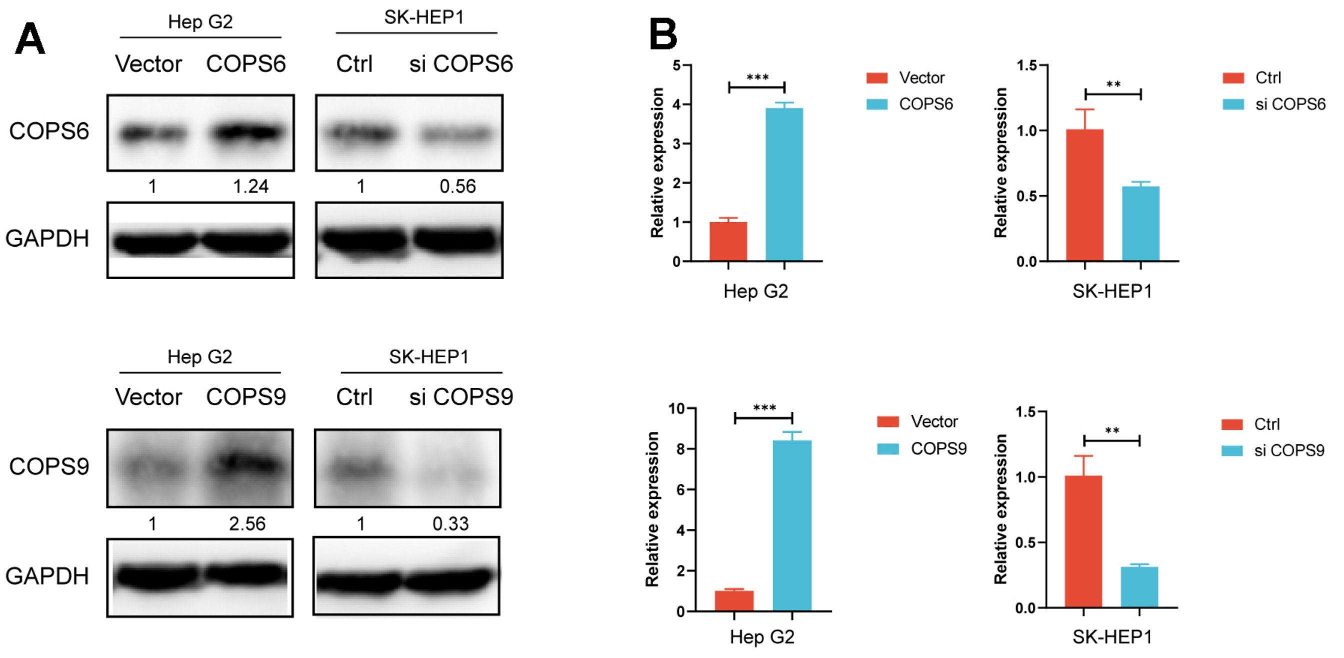

Supplementary Figure 3. Efficiencies of COPS6/9 overexpression and knockdown in Hep G2 and SK-HEP1 cells following plasmid and siRNA transfection, respectively, as confirmed by Western blotting (**A**) and qPCR (**B**). **p < 0.01, ***p < 0.001.
